# p53 Activation in Genetic Disorders: Different Routes to the Same Destination

**DOI:** 10.3390/ijms22179307

**Published:** 2021-08-27

**Authors:** Yu-Young Tsai, Chun-Hao Su, Woan-Yuh Tarn

**Affiliations:** Institute of Biomedical Sciences, Academia Sinica, Taipei 11529, Taiwan; wesleytsai1999@ibms.sinica.edu.tw (Y.-Y.T.); chsu@ibms.sinica.edu.tw (C.-H.S.)

**Keywords:** centrosome, developmental disorders, DNA damage repair, p53, ribosome, telomere

## Abstract

The tumor suppressor p53 is critical for preventing neoplastic transformation and tumor progression. Inappropriate activation of p53, however, has been observed in a number of human inherited disorders that most often affect development of the brain, craniofacial region, limb skeleton, and hematopoietic system. Genes related to these developmental disorders are essentially involved in transcriptional regulation/chromatin remodeling, rRNA metabolism, DNA damage-repair pathways, telomere maintenance, and centrosome biogenesis. Perturbation of these activities or cellular processes may result in p53 accumulation in cell cultures, animal models, and perhaps humans as well. Mouse models of several p53 activation-associated disorders essentially recapitulate human traits, and inactivation of p53 in these models can alleviate disorder-related phenotypes. In the present review, we focus on how dysfunction of the aforementioned biological processes causes developmental defects via excessive p53 activation. Notably, several disease-related genes exert a pleiotropic effect on those cellular processes, which may modulate the magnitude of p53 activation and establish or disrupt regulatory loops. Finally, we discuss potential therapeutic strategies for genetic disorders associated with p53 misactivation.

## 1. Introduction

The p53 gene is most frequently altered in human malignancies, indicating its biological and clinical importance [1]. p53 transactivates approximately one hundred target genes that exert diverse biological functions—primarily cell cycle regulation, DNA repair, and apoptosis [2,3]. p53 also represses gene expression via the action of its downstream targets, such as p21 [3]. Given the role of p53 in tumor suppression, *Trp53* ablation induces tumors in mice at an early age [4]. Moreover, *Trp53* knockout mice exhibit developmental abnormalities, which, however, differ between strains of mice with different genetic backgrounds, indicating that p53 contributes to cell differentiation and development [5]. p53 also participates in various metabolic pathways and coordinates metabolic homeostasis; dysregulation of p53 function may lead to metabolic disorders and perhaps tumorigenesis [6]. Moreover, p53 regulates cell senescence and aging through multiple signaling pathways [7]. In response to cellular stress(es) of different types or intensities, p53 may induce transient cell-cycle arrest to allow damage repair or evoke senescence or apoptosis that, respectively, halts damage propagation and eliminates damaged cells [8]. Abnormal and/or persistent p53 activation promotes tissue degeneration [9]. For example, excess p53-induced neuronal death is linked to neurodegenerative diseases such as Alzheimer’s disease [9]. Additionally, excessive p53 activation during embryonic development has been observed in a variety of congenital disorders [10]. In this review, we discuss several sets of genetic mutations that contribute to excessive p53 activation, leading to phenotypic abnormalities in congenital disorders.

## 2. Molecular Function and Regulation of p53

p53 is a master regulator of cell-fate determination in response to cellular stress or DNA damage, primarily via its role in transcriptional regulation. Upon genotoxic or oncogenic insults, p53 activates the expression of genes involved in DNA damage repair, cell-cycle arrest, apoptosis, senescence, and autophagy [11,12]. p53 directly participates in various DNA repair pathways to maintain genome stability [13]. In addition, cytoplasmic p53 can inhibit autophagy and promote apoptosis or necrosis [14,15]. Deregulation of p53 function may lead to metabolic dysfunction, aging, or tumorigenesis [6]. In human cancer, mutant p53 in general loses its ability to suppress tumorigenesis and may, however, gain oncogenic potential such as promoting tumor cell survival or adaptation to stress [16,17].

**Figure 1:**
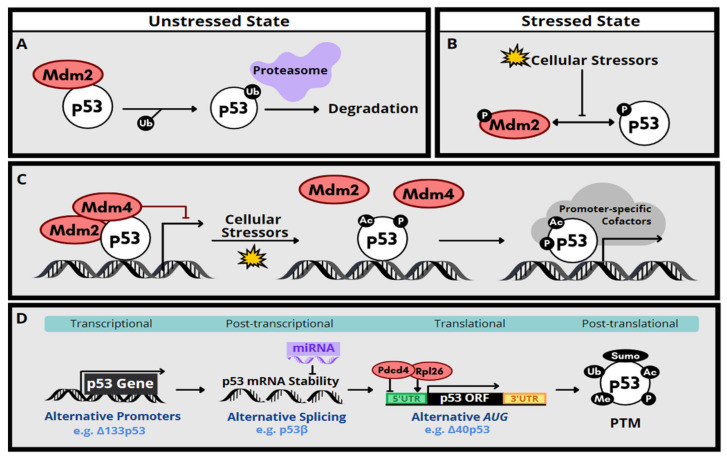
Regulation of p53 gene expression and protein stability/activity. (**A**) In unstressed cells, p53 is subjected to Mdm2-mediated ubiquitination, followed by proteosome-dependent degradation. (**B**) Under stressed conditions, phosphorylation of Mdm2 an p53 prevents their interaction, thereby stabilizing p53. (**C**) Cellular stressors, such as DNA damage, may relieve p53 from Mdm2/Mdm4-mediated suppression from promoters. For full transcriptional activation of p53-responsive genes, p53 may undergo various post-translational modifications and recruit promoter-specific cofactors. (**D**) Depicted are additional molecular mechanisms by which p53 gene/protein expression is regulated. Abbreviations: UTR, untranslated region; ORF, open reading frame.

The expression of p53 is regulated by multiple signaling pathways and mechanisms. In non-stressed cells, p53 level is restricted primarily by Mdm2-mediated ubiquitination and proteasomal degradation, thereby constraining its anti-proliferation and pro-apoptotic functions [18] (Figure 1A). However, DNA damage or oxidative stress results in phosphorylation of Mdm2 and p53, leading to disruption of the Mdm2-p53 interaction. The level of p53 protein is consequently increased (Figure 1B). In addition to the ubiquitination activity toward p53, Mdm2 can suppress the transactivation activity of p53 [19]. DNA damage induces acetylation of p53 and releases p53 from repression by Mdm2 or its homolog Mdm4, leading to activation of p53-responsive target genes [18] (Figure 1C). Moreover, additional phosphorylation/dephosphorylation events and various types of post-transcriptional modifications such as acetylation and SUMOylation can also modulate p53 stability or activity [18,19] (Figure 1D).

Besides post-translational modification, cellular p53 level can be regulated via mRNA stability or translational control [20]. A large set of miRNAs act as negative regulators of p53 by promoting mRNA degradation [21]. In response to DNA damage, p53 mRNA translation is activated by ribosomal protein L26 (Rpl26) or derepressed from programmed cell death protein 4 (Pdcd4)-mediated suppression [22,23] (Figure 1D). Moreover, p53 isoforms that are generated by alternative transcriptional initiation, alternative splicing, or alternative translation initiation have been detected in various types of cancer or can be induced in response to cellular stimuli [24] (Figure 1D). Those *N*- or *C*-terminally truncated p53 isoforms may positively or negatively modulate the transcriptional activity of p53 or act independently. Certain p53 isoforms, such as ∆40p53, are relatively more stable [25]. Co-expression of wild-type or mutant p53 isoforms in cancer cells, however, results in diverse effects on tumorigenesis [26]. Finally, some of the p53 isoforms contribute to embryonic development, neurodegeneration and inflammation, indicating their physiological and pathological significance [27,28,29].

## 3. p53 Activation Associated with Congenital Anomalies

Studies of gene mutants or knockouts in mouse models have revealed that inappropriate p53 activation accounts for different extents of developmental defects [30]. Phenotype severity correlates with the degree of p53 activation. For example, homozygous knockout of *Mdm2* results in early embryonic lethality [31]. As compared to *Mdm2*-null mice, *Mdm2*^puro^ heterozygotes express modest levels of Mdm2 and hence exhibit mild p53 activation. These mice were alive, albeit with a short lifespan and hematopoietic defects [32]. Some p53 mutants display a higher stability or activity. Mice of strains with such mutant p53 die at different embryonic stages with tissue-specific defects, or have reduced lifespan and premature aging phenotypes [10]. For example, the p53^7KR^ mutant bearing mutations in the *C*-terminal acetylation/ubiquitination sites has higher basal expression and transactivation activity towards the cell-cycle inhibitor p21 than does wild-type p53 [33]. The p53^25,26,53,54^ mutant by itself is transcriptionally inactive due to mutations in the transactivation domains, but it can stabilize and hence upregulate wild-type p53 [34]. The increase in p53 protein, either by stabilizing p53 or by inactivating Mdm2/4, causes diverse phenotypes—from embryonic lethality to developmental defects such as craniofacial malformation, microcephaly, and reduced bone marrow cellularity [10]. It is possible that excessive p53 activity inhibits proliferation and/or induces apoptosis of stem/progenitor cells, resulting in hypoplasia. A recent study using mouse models demonstrates that the cellular level as well as the spatiotemporal expression pattern of p53 determines the nature and severity of developmental syndromes [10].

Similar to the above mouse models, a variety of human congenital disorders arises from inappropriate activation of wild-type p53. First described in 1900, the Treacher Collins syndrome (TCS) is a rare genetic disorder that exhibits multiple facial dysmorphisms [35]. *TCOF1* is the primary gene associated with TCS, encoding an RNA polymerase I associated factor that regulates ribosome biogenesis [35]. *Tcof1* deficiency results in decreased proliferation of both neural ectoderm and neural crest cells, and this underlies the observed craniofacial anomalies in TCS [36]. Nucleolar stress triggers stabilization and activation of p53, whereas inhibition of p53 activity prevents craniofacial maldevelopment, providing a pathological link between ribosomal defects and aberrant p53 activation [37,38]. Excessive p53 activation is a notable contributor to human disorders of ribosome dysfunction, namely ribosomopathies such as Diamond Blackfan anemia (DBA) and 5q^–^ syndrome [39,40]. Analogous to ribosomopathies, several congenital disorders also exhibit p53 upregulation, including Fanconi anemia (FA, with genetic defects in DNA damage repair and response), dyskeratosis congenita (DC, telomere replication defects), and primary microcephaly (centrosome duplication defects) [41,42,43]. Because disruption of any of these cellular activities may result in chromosome and/or genome instability, p53 activation is a conceivable consequence. Generally, loss of p53 can rescue—at least to some extent—the phenotypes of mouse models of p53 activation-associated human congenital disorders, indicating that excessive p53 leads to congenital abnormalities [10]. In addition to the aforementioned disorders, mutation of several tissue-specific transcription or chromatin remodeling factors, namely Chd7, Tbx1, and Pax3, is also linked to p53 activation-associated disorders such as CHARGE, 22q11.2 deletion, and Waardenburg syndromes, respectively. We refer interested readers to recent reviews [44,45,46].

## 4. Genetic Disorders Related to Excessive p53 Activation

### 4.1. Disorders of Ribosome Dysfunction

**Figure 2:**
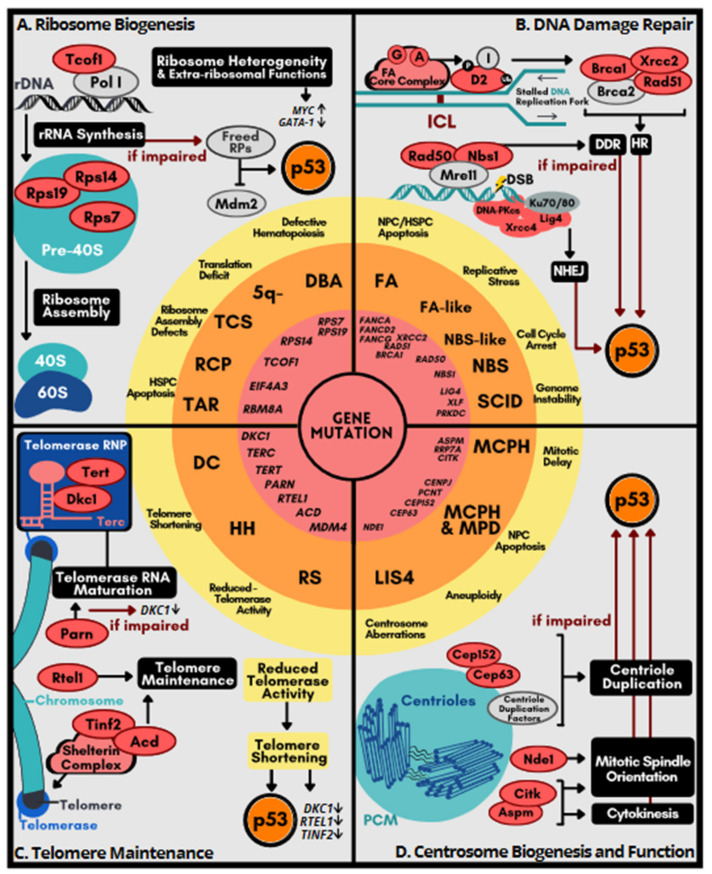
Cellular processes implicated in p53 hyperactivation-associated genetic disorders. Genetic alterations (mutation/deletion; red circle in the center) lead to their respective human congenital disorders (orange zone) via disruption of various cellular processes or activities, including ribosome biogenesis (**A**), DNA damage repair (**B**), telomere maintenance (**C**), and centrosome duplication (**D**) as depicted in the four quadrants. p53 hyperactivation as a common feature shared across these genetic aberrations in murine models is depicted in the most center of this figure. The resulting molecular and cellular defects (yellow zone) underlie each group of disorders. Red ovals indicate the encoded proteins that, when dysregulated or absent, contribute to the syndrome discussed in the text. Hyperactive p53 is represented by a bold red-filled circle, as opposed to p53 at baseline in white-filled circle. Abbreviations: RCP, Richieri-Costa–Pereira; RS, Revesz syndrome; LIS4, Lissencephaly-4.

Ribosome biogenesis is a major determinant of translational capacity. Neoplastic transformation requires upregulation of both ribosome biogenesis and translation, whereas certain congenital diseases result from defects in ribosome assembly. DBA syndrome, which is characterized by chronic macrocytic–normocytic anemia, is a typical example of ribosomopathies [39,47]. Approximately 20 DBA-associated genes encode ribosomal proteins (RPs) or factors that participate in rRNA processing or ribosomal biogenesis [39,40] (Figure 2A). For example, mutations in *RPS19* have been identified in ~25% of affected individuals. *Rps19* knockout mice develop macrocytic anemia, as observed in DBA patients [48]. *Rps19* deficiency impairs cell proliferation and induces apoptosis of hematopoietic progenitors. Genetic ablation of p53 can help restore the normal phenotype, and the effect depends on the extent of ablation. Analogously, haploinsufficiency of *Rps14*, a causal factor in 5q^–^ syndrome, also leads to macrocytic anemia [49]. Knockout of p53 rescues the defect in bone marrow progenitor cells. As described above, mutations in rDNA transcription factors such as *TCOF1* that are linked to TCS also cause p53 activation [38]. Therefore, excess p53 activation, secondary to ribosome deficiency, accounts for the depletion of hematopoietic stem/progenitor cells (HSPCs). Inactivation of p53 alleviates the pathobiology of ribosomopathies.

Disruption of ribosome biogenesis as a consequence of RP mutations or impaired rRNA synthesis results in free RPs that are not incorporated into ribosomes. Unincorporated RPs can bind to and inhibit Mdm2, thereby increasing the half-life of p53 (Figure 2A). Increased p53 activity can trigger cell apoptosis [50]. The extra-ribosomal functions of certain RPs may also contribute to pathogenesis of ribosomopathies [51,52]. For example, Rpl11 destabilizes *c-Myc* mRNA by recruiting the RNA-induced silencing complex, leading to suppression of *c-Myc* target genes [53,54]. Therefore, *c-Myc* activity is likely upregulated in *Rpl11*-deficient cells. Accordingly, *Rpl11* haploinsufficient adult mice have increased susceptibility to lymphomagenesis under genotoxic stress in part by upregulating *c-Myc* [55]. A notable feature of ribosomopathies is the tissue-specificity of clinical presentation. One explanation is that reduced ribosome levels may selectively compromise the translation of certain cell-type specific mRNAs [51,52]. For example, the translation of GATA1, a master transcription factor for hematopoietic genes, is particularly impaired in RP-deficient DBA cells, perhaps owing to secondary structures in its 5′ untranslated region [56]. A follow-up study revealed that reduced ribosome abundance in hematopoietic cells with DBA-associated lesions indeed impairs the translation of a set of master regulators for erythroid differentiation [57]. Another possible explanation for tissue specific phenotypes of ribosomopathies is ribosome heterogeneity, in which RP composition or rRNA modification is altered [58,59]. Ribosome heterogeneity may contribute to specialized translation, resulting in lineage-biased protein expression. In general, ribosomal defects alter the cellular translational landscape and rewire metabolic programs, shifting cells towards an oncogenic state. Therefore, ribosome heterogeneity/specialization provides an explanation of how congenital ribosomopathies display hypoproliferative anemia during infancy and early childhood and have an increased cancer risk later in life [51,52].

The two genes, *EIF4A3* and *RBM8A*, encoding nonsense-mediated mRNA decay factors, have been implicated in Richieri-Costa–Pereira syndrome and thrombocytopenia absent radius (TAR) syndrome, respectively [60,61,62]. Heterozygous knockout of either gene in the mouse neocortex causes microcephaly, accompanied by p53 activation; knockout of p53 allows for a partial rescue of cortical development [63,64]. TAR syndrome is characterized by the absence of the radius bone and thrombocytopenia. Accordingly, *Rbm8a* ablation in megakaryocyte precursors severely reduces the platelet count in mice [65]. It is possible that elevated p53 impairs polyploidization of megakaryocytes. p53 knockout partially restored the level of platelets in *Rbm8a* knockout, supporting that TAR is also a p53 activation-related disorder. Notably, transcriptome analysis reveals a reduction in the expression of ribosomal protein mRNAs, indicating that *Rbm8a* deficiency may compromise ribosome biogenesis [64]. *Rbm8a* deficiency also results in centrosome aberrations and DNA damage accumulation [66,67,68], which have the potential to induce p53. In addition, *RBM8A* (Y14) depletion inhibits ubiquitination of p53 and thus stabilizes p53 in cell cultures [69]. Therefore, *RBM8A* deficiency may induce p53 activation via multiple pathways. Finally, given that TAR syndrome results from a deletion of a ~200 kb 1q21.1 region that encompasses *RBM8A* as well as another 15 genes, whether and how haploinsufficiency of these genes contributes to TAR pathogenesis remains obscure.

### 4.2. Disorders Related to DNA Repair Deficiency

DNA repair defects cause a broad spectrum of clinical phenotypes, including neurological disease, accelerated aging, and cancer predisposition. In addition, deficiency in certain DNA repair pathways, i.e., the FA pathway, double-strand DNA break (DSB) repair and DNA damage response (DDR), is particularly associated with inherited developmental disorders [70].

The FA pathway is involved in the repair of DNA interstrand crosslink (ICLs), which generally cause replication arrest and may also give rise to DSBs [71] (Figure 2B). The FA complex consisting of ~20 FA factors detects stalled replication forks and triggers the ATR-Chk1 cell-cycle checkpoint along with a series of reactions involving post-translational modifications of FA factors to resolve ICLs [72,73]. The FA pathway ensures repair fidelity for ICL-derived DSBs by promoting homologous recombination (HR) over the competing non-homologous end joining (NHEJ) repair pathway. Notably, *BRCA1/2* (Fancs/Fancd1) play a critical role in HR. The FA/BRCA pathway also resolves DNA replication stresses induced by transcription–replication collision, DNA-RNA hybrids, or G-quadruplexes [74]. Dysfunction of FA-associated pathways leads to the accumulation of DNA damage with consequent increased chromosomal instability.

The majority of FA patients develop bone marrow failure; among them, more than half suffer from congenital defects such as microcephaly and skeletal abnormalities [75]. Bone marrow failure results from progressive impairment of HSPCs [76]. Constitutive p53 induction is observed in FA fibroblasts [77]. *Fancd2* knockout in mice induces G0/G1 cell-cycle arrest in HSPCs, which can be rescued by knockdown of p53 or p21, indicating that an elevated p53/p21 response impairs hematopoiesis [77]. It is likely that, during fetal development of FA-afflicted subjects, excess p53 activation that results from replicative stress diminishes the pool of HSPCs, leading to bone marrow failure. Notably, *Fanca* or *Fancg* knockout reduces the capacity for NPC self-renewal during developmental and adult neurogenesis [78]. Conceivably, excessive p53 accounts for aging-associated neural stem cell exhaustion in such FA mouse models. Mutations in the genes that are involved in both the FA and HR pathways such as *BRCA1*, *RAD51* and *XRCC2* confer susceptibility to cancer [70]. Biallelic mutations in any of these genes cause embryonic lethality that can be partially rescued by p53 knockout [79,80,81], indicating that reduction of p53 may restore cell cycle and/or prevent cell death.

DSB repair involves HR or NHEJ. Besides the aforementioned HR/FA factors, defective NHEJ genes are also linked to congenital disorders (Figure 2B). Mutations in *LIG4*, *XLF* or *PRKDC* cause profound immunodeficiency and/or microcephaly [82]. NHEJ plays a critical role in lymphocyte development by resolving the programmed DSBs generated during V(D)J recombination. Therefore, NHEJ defects compromise the immune system. As observed in FA genes, knockout of any of the NHEJ genes (*Lig4*, *Xrcc4* or *Prkdc*) causes excessive neuronal apoptosis and this defect can be rescued by co-deletion of the p53 gene [83,84,85]. The Mre11-Rad50-Nbs (MRN) complex has multiple functions in DDR, including DSB recognition, DNA replication fork stabilization, and telomere maintenance [86] (Figure 2B). Mutations in *NBS1/NBN* and *RAD50*, respectively, cause Nijmegen breakage syndrome (NBS) and an NBS-like disorder, with consequent microcephaly, but only the former suffers from immunodeficiency [87]. Knockout of *Nbn* in the central nervous system results in microcephaly as well as cerebellar ataxia, which can be rescued by p53 ablation [88].

In conclusion, DNA repair impairment causes genome instability and activates p53-mediated cell-cycle arrest or apoptosis that constrains the size of the hematopoietic/neural stem cell pools, leading to bone marrow failure or microcephaly. Finally, it is noteworthy that an increased p53 activity attenuates the FA DNA repair pathway and hence exacerbates DNA damage, suggesting a positive feedback loop [89].

### 4.3. Syndromes Caused by Telomere Dysfunction

Telomeres protect the ends of eukaryotic chromosomes to prevent chromosomes from fusion and degradation, thus maintaining chromosome length and therefore genome integrity [90]. Telomere shortening is associated with aging. On the other hand, upregulation of the telomere maintenance mechanism is a common feature of cancer [91]. Telomeres consists of thousands of hexameric TTAGGG repeats, and telomere replication is catalyzed by the telomerase, a ribonucleoprotein complex containing the RNA template *Terc*, the reverse transcriptase Tert, and several additional factors including the pseudouridine synthase Dyskerin (Dkc1). Dkc1 binds to the H/ACA box of the telomerase RNA, which is a hairpin structure also characteristic of a class of small RNAs in the nucleolus or Cajal bodies [92]. In addition, the shelterin complex, which consists of six different subunits, binds to both double-stranded and single-stranded telomeric repeats and also plays a critical role in maintaining chromosome stability by preventing hyper-resection at telomeres (Figure 2C).

Telomere disorders (also called telomeropathy syndromes) are characterized by genetic deficits in telomere maintenance, including the archetypal DC syndrome and its variants such as Hoyeraal Hreidarsson syndrome and Revesz syndrome. DC exhibits high rates of bone marrow failure as well as other symptoms such as cutaneous pigmentation and nail hypertrophy [41]. DC is caused by germline mutations of genes that participate in telomere biogenesis, including telomerase (*DKC1*, *TERC*, *TERT*), the shelterin complex (*TIN2*, *TPP1*), and telomere elongation helicase *RTEL1* [41] (Figure 2C). The most prevalent mutations in DC occur in *DKC1*, *TERC*, *TERT*, and *TIN2*. Dkc1 regulates the accumulation of the human telomerase RNA. DC-associated mutations of *DKC1* not only cause destabilization of telomerase RNA and telomere shortening but also impair ribosome biogenesis [92]. *Dkc1* knockout results in activation of the p53-dependent cell-cycle checkpoint pathway [93]. DC-associated mutations in three other telomerase or shelterin genes, namely *TERC*, *TERT*, and *TIN2*, also impair telomere integrity and induce p53 expression [94]. *Terc* knockout-induced telomere shortening impairs adult neurogenesis in the lateral ventricles of the brain; p53 ablation rescues such defects [95]. Knockout of the telomere shelterin component *Acd/Tpp1* in epidermal keratinocytes results in skin hyperpigmentation and impairs hair-follicle morphogenesis, both of which are also reversed by p53 knockout [96].

It is intriguing that reduced telomerase activity or telomere shortening has been observed in defects of a number of non-telomerase factors. For example, the poly(A)-specific ribonuclease PARN is involved in mRNA turnover by degrading the poly(A) tail of mRNAs. Hoyeraal Hreidarsson syndrome-associated mutations in *PARN* result in telomere shortening [97]. *PARN* deficiency downregulates telomerase RNA and several transcripts encoding telomere-related factors, hence compromising telomerase activity [97]. A missense mutation of *MDM4* (T454M) has been identified in individuals with DC-like phenotypic traits [98]. However, homozygous *Mdm4*^T454M^ mutation causes neonatal death, but embryonic fibroblast cells derived from *Mdm4*^TM/TM^ mice exhibit increased p53 level and decreased telomere length, a cardinal feature of telomeropathies [98]. Homozygous p53 mutant (p53^∆31/∆31^) mice express a *C*-terminally truncated and hyperactive form of p53 phenocopy telomere disorder syndromes. Telomeres in such p53^∆31^ mutant cells are significantly shorter than those in wild-type cells [99]. It appears that p53 activation downregulates the expression of several genes involved in telomere metabolism, including *Dkc1*, *Rtel1*, and *Tin2* [99,100], but how this regulation achieves gene specificity remains unclear. Collectively, severe telomere shortening activates p53, which may further compromise telomerase activity, suggesting a regulatory loop which exacerbates telomere shortening.

### 4.4. Disorders Related to Centrosome Dysfunction

The centrosome is an evolutionarily conserved cylindrical organelle that acts as the main microtubule-organizing center to direct bipolar mitotic spindle assembly for accurate segregation of chromosomes during cell division. The centrosome contains two centrioles in orthogonal configuration surrounded by pericentriolar material that harbors proteins responsible for microtubule nucleation and anchoring and regulating cell-cycle checkpoints [101] (Figure 2D). Centriole duplication is coupled with the cell cycle in a highly spatiotemporally regulated manner [101]. Upon exit from mitosis, centriole pairs disengage and duplicate at the G1/S transition, and subsequently centrosomes become mature and separated. By metaphase, centrosomes move to opposing poles of the cell to dictate the organization of the mitotic spindles for chromosome separation. Deregulation of centrosome biogenesis leads to structural or numerical aberrations of centrosomes [102]. Centrosome abnormalities are observed in most cancer types, which may result from deregulation of oncogenes or tumor suppressors or perturbation of cell cycle progression. Centrosome aberrations may consequently cause chromosomal instability and perturb asymmetric cell division. Notably, centrosome aberrations can trigger an immunosuppressive microenvironment and promote dissemination of metastatic cells [103,104], emphasizing the impact of centrosome abnormalities in tumorigenesis.

A large set of centrosomal factors participate in centriole duplication and stability control, γ-tubulin recruitment and centrosomal attachment of the γ-tubulin ring complex [105]. Mutations in these centrosome-related genes cause developmental defects—primarily microcephaly and also growth failure [106]. Among disorders induced by centrosome aberrations, microcephaly primary hereditary (MCPH) is the most extensively studied. Notably, nine of the 27 *MCPH*-associated genes are implicated in centriole biogenesis and/or the coupling of the centrosome cycle and mitosis [107] (Figure 2D). *ASPM* (*MCPH5*) mutations represent the most common cause of *MCPH*. Aspm, which localizes to centrosomes and spindle poles, is required for spindle organization and positioning [108]. *Aspm* knockout in cerebellar granule neuron progenitors alters cell-division orientation and induces premature differentiation and apoptosis. Knockout of *Bax* or p53 restores cerebellar growth of *Aspm*-deficient mice [109]. Aspm functions coordinately with the citron Rho-interacting kinase CitK to regulate mitotic spindle orientation [110]. Notably, loss or inactivation of *CitK* also causes primary microcephaly and p53 ablation mitigates such phenotypes [111], suggesting that *CitK* deficiency causes p53-dependent microcephaly. In addition, mutations of several other centrosome-related genes, such as *PCNT*, *CENPJ*, *CEP152*, and *CEP63*, are linked not only to *MCPH* but also to Seckel syndrome, characterized by dwarfism in addition to microcephaly [112]. Cep63 localizes to the centrosome but dissociates upon DNA damage. Cep63 and Cep152 act in concert to ensure efficient centriole duplication [113]. Cep63 deficiency compromises centriole duplication, leading to mitotic spindle defects in NPCs. Hence, delayed mitosis triggers p53-induced apoptosis, as observed in *Aspm* knockout mice; knockout of p53 restores the number of NPCs [114].

*Nde1* is a candidate gene for lissencephaly-4, which is characterized by both microcephaly and lissencephaly. *Nde1* is a centrosomal factor that regulates mitotic spindle assembly and also is necessary for dynein function [115] (Figure 2D), which contributes to interkinetic nuclear migration and mitosis of radial glial cells. Therefore, *Nde1*-null mice exhibit severe microcephaly. *Nde1* mutant NPCs undergo p53-dependent apoptosis owing to catastrophic DNA damage, leading to neuron depletion in the middle cortical layers [105]. p53 depletion is sufficient to restore cortical development and brain size in knockout mice.

In summary, mutations in centrosome-related genes can result in defective centriole duplication or spindle orientation in NPCs, leading to apoptosis of progeny cells. A recent report indicated that centrosome defects in NPCs cause delayed mitosis, which triggers a mitotic surveillance pathway [116]. Consequently, p53-dependent apoptosis of NPCs reduces cortical expansion and hence causes microcephaly.

**Figure 3:**
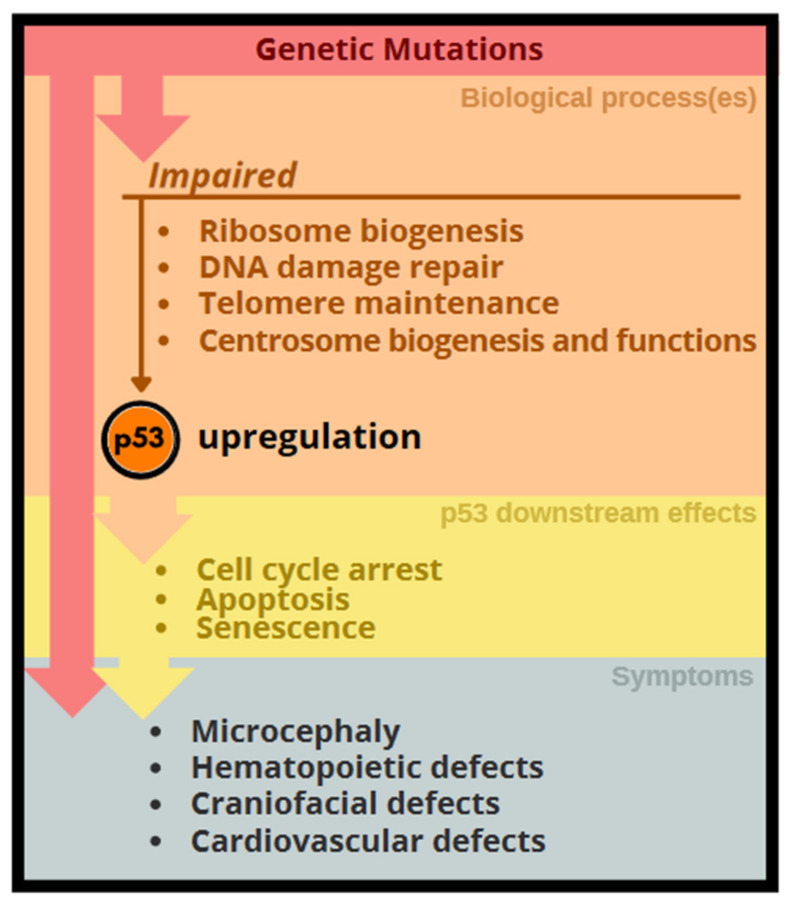
Genetic impact activates the p53 networks resulting in developmental abnormalities. Genetic alterations that disrupt several different cellular processes as depicted lead to upregulation of p53 networks, which leads to stem cell deficiency-associated phenotypic abnormalities. Gene mutations also have direct impact on phenotypes.

In conclusion, dysregulation of the aforementioned yet different cellular processes during development commonly lead to stabilization or induction of p53. A high level of p53 in general induces cell cycle arrest and/or apoptosis of various stem and progenitor cells, and hence result in phenotypic abnormalities, such as microcephaly, bone marrow failure, and craniofacial deformation, in respective genetic disorders (Figure 3).

## 5. Interrelationships of the Cellular Processes Implicated in p53 Activation-Associated Disorders

**Figure 4:**
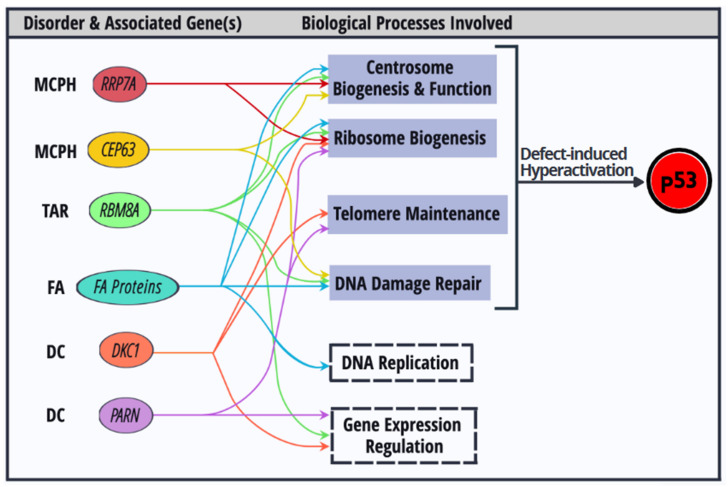
Dysregulation of biological processes that activate p53 and their interconnection. Colored ovals depict protein factors encoded by congenital disorder-associated genes. These factors participate in multiple biological processes. Dysregulation of each biological process (blue grey) activates p53.

Several aforementioned disease genes may exert pleiotropic effects on different cellular processes (Figure 4). For example, *Dkc1* mutations not only cause telomere shortening, but also impair ribosome biogenesis and subsequently compromise mRNA translation [117,118]. Notably, *PARN* deficiency particularly downregulates the transcripts encoding factors involved in telomere maintenance, including *Dkc1* [97]. Therefore, impaired rRNA biogenesis is observed in *PARN*-mutated or knockout cells. Perhaps compromised translation resulting from defective rRNA modification/processing can explain the tissue-specific symptoms and cancer predisposition found in DC patients [119]. The recently identified MCPH gene *RRP7A* encodes an 18S rRNA processing factor. Intriguingly, Rrp7a also localizes to the centrosome and cilia. MCPH-derived dermal fibroblasts display defects in rRNA processing and ciliary dynamics. *Rrp7a* knockout zebrafish exhibit microcephaly-like phenotypes [120]. Thus, it would be interesting to know whether and how Rrp7a links centrosome dynamics and rRNA biogenesis during cortical development, and whether p53 inactivation is sufficient to revert phenotypes caused by *Rrp7a* deficiency. Centrosome biogenesis is tightly controlled throughout the cell cycle and is sensitive to DNA damage. After DNA damage, DDR kinases ATM/ATR delocalize Cep63 from the centrosome to prevent centrosome-dependent microtubule assembly [121]. Abnormal centrosome numbers are frequently observed in fibroblasts of FA patients. Indeed, several FA proteins localize to the mitotic apparatus during cell division and help ensure the fidelity of chromosome segregation [122]. Inactivation of the FA pathway leads to spindle checkpoint failure and induces supernumerary centrosomes. Recent reports have indicated that Fanca and Fanci play a role in ribosome biogenesis or nucleolar homeostasis, suggesting a potential link between DNA damage and nucleolar stress responses [123]. In addition to mRNA surveillance, *RBM8A* participates in DNA damage repair and centrosome organization [66,67,68]. It is conceivable that several critical cellular processes that span genome integrity, mRNA/protein expression, and cell cycle/division, are interrelated to ensure proper cellular function.

Finally, it is plausible that multiple defective processes that result from a single mutant gene converge on p53 signaling; consequently, differential degree of p53 activation may influence the tissue specificity of cellular defects.

## 6. Therapeutic Strategies for p53 Activation-Associated Disorders

As demonstrated by using animal models, p53 activation-induced developmental defects can be rescued, albeit often partially, by concomitant deletion of p53. This possibility makes pharmacological attenuation of hyperactive p53 an attractive therapeutic strategy. Pifithrin-α is thought to inhibit p53-dependent transactivation; however, it prevents DNA damage-induced apoptosis, mitochondrial damage, and caspase activation, likely via multiple mechanisms [124]. Pifithrin-α restores erythroid differentiation of Rps14/19-depleted CD34^+^ hematopoietic stem cells in vitro, indicating its potential in reversing the erythropoietic defects in DBA or 5q^–^ syndrome [125]. A recent report revealed that the FDA-approved calmodulin inhibitor trifluoperazine improves erythropoiesis in animal models of DBA by suppressing p53 mRNA translation [126]. Therefore, suppressing the expression or activity of p53 confers therapeutic value for p53 hyperactivation-associated disorders. siRNA-based p53 silencing may also be one of the future therapeutic approaches [127].

Besides direct inhibition of p53, a number of therapeutic strategies have been developed to target each cellular process discussed above. l-leucine upregulates mTOR signaling, subsequently promoting ribosome biogenesis and global translation via different molecular mechanisms. Administration of l-leucine improves anemia and increases bone marrow cellularity, accompanied by downregulation of p53 activity, in animal models of DBA [128,129]. Treatment with an antioxidant ameliorates craniofacial abnormalities by reducing the levels of DNA damage-induced reactive oxygen species in *Tcof1*-deficient animal models of TCS [130]. In addition, *Tcof* deficiency reduces the abundance of Cnbp/Znf9, which is an RNA binding protein required for the expansion of neural crest cells [131]. In a zebrafish model of TCS, inhibition of proteasomes can attenuate craniofacial malformations by restoring the level of Cnbp/Znf9 [132]. Treatment with danazol, an androgen derivative that aromatizes into estrogens, can upregulate *TERT* via nuclear receptors and hence promotes telomere elongation in DC patients [133]. Hyperactivated TGF-β signaling contributes to the suppression of hematopoiesis in bone-marrow failure disorders such as FA and myelodysplastic syndrome [134,135]. Therefore, pharmacological agents that inhibit TGF-β signaling constitute a potential therapeutic option for FA. Moreover, transducing gene-corrected autologous hematopoietic stem cells in patients is also a potential therapeutic strategy [136].

## 7. Conclusions and Outlook

Understanding the genetic causes and molecular mechanisms underlying the aforementioned congenital disorders may inform the development of therapeutic strategies. Over the past two decades, studies using tissue/cell-specific knockout mice have revealed that disruption of certain cellular processes can upregulate p53 and recapitulate developmental defects observed in the corresponding human disorders. In general, excess p53 restrains cell proliferation and/or induces apoptosis of various stem/progenitor cells during embryonic and/or postnatal development. However, many challenges remain in the quest to improve our understanding of p53 activation-associated disorders.

First, p53 suppression often partially rescues the mutant phenotypes of animal models of the aforementioned disorders, indicating that individual disorder-related factors may have specialized roles in cellular functions and development. Moreover, it must be noted that murine p53 isoforms are similar but not identical to their human counterparts. For example, depletion of TAR syndrome-associated *RBM8A* induces the isoform p53β in human cells, which is absent in mice [69]. Therefore, the phenotypes of various disorders may be differentially affected by expression of the different p53 isoforms that are produced by humans or mice.

Second, it is important to know how mutations in different components of a macromolecular machine—such as the ribosome or centrosome—result in specific phenotypes besides the common ones. Therefore, future investigation should aim to reveal how different cell types confer differential tolerance to dysregulation of a certain cellular process. For example, to understand how ribosome heterogeneity contributes to the regulation of the proteome in various cell types, knock-in tagging experiments with a wild-type or mutant RPs (followed by Ribo-seq analysis) may help to reveal cell type-specific translatomes [137].

Finally, regarding chromosome deletion syndromes such as 5q^−^ syndrome (5q33.1 deletion) and TAR syndrome (1q21.1 deletion), it is important to decipher how co-deleted genes contribute to pathogenesis. For example, different mouse models of 5q^−^ syndrome have been generated via deletion of a large chromosome interval syntenic to human 5q33.1, including *Rps14*, or co-deletion of *Rps14* with three other 5q^−^ syndrome genes (*Csnk1a1/miRNA145/miRNA146a*) [49,138]. These mice recapitulate the features of 5q^−^ syndrome to different extents. Therefore, chromosome engineering, combinatorial gene deletions, and knock-in strategies will greatly facilitate the generation of mouse models that closely mimic human genetic disorders.

## Data Availability

Not applicable.

## Abbreviations

5q^−^: 5q^−^ syndrome; DBA: Diamond Blackfan anemia; DC: dyskeratosis congenita; DDR: DNA damage response; DSB: double-strand DNA break; FA: Fanconi anemia; HH: Hoyeraal Hreidarsson syndrome; HR: homologous recombination; HSPC: hematopoietic stem/progenitor cells; ICL: interstrand crosslink; LIS4: lissencephaly-4; NBS: Nijmegen breakage syndrome; NHEJ: non-homologous end joining; NPC: neural progenitor cells; MCPH: microcephaly primary hereditary; MPD: microcephalic primordial dwarfism; PCM: pericentriolar material; RCP: Richieri-Costa–Pereira syndrome; RNPs: ribonucleoprotein particles; RPs: ribosomal proteins; RS: Revesz syndrome; SCID: severe combined immunodeficiency; TAR: thrombocytopenia absent radius; TCS: Treacher Collins syndrome.
